# An fNIRS investigation of novel expressed emotion stimulations in schizophrenia

**DOI:** 10.1038/s41598-023-38057-1

**Published:** 2023-07-10

**Authors:** Cuiyan Wang, Yueqian Zhang, Lam Ghai Lim, Weiqi Cao, Wei Zhang, Xiaoyang Wan, Lijun Fan, Ying Liu, Xi Zhang, Zengjie Tian, Xiaojun Liu, Xiuzhi Pan, Yuan Zheng, Riyu Pan, Yilin Tan, Zhisong Zhang, Roger S. McIntyre, Zhifei Li, Roger C. M. Ho, Tong Boon Tang

**Affiliations:** 1 Huaibei Normal University, Huaibei, China; 2 Huaibei Mental Health Center, Huaibei, China; 3 Department of Electrical and Robotics Engineering, School of Engineering, Monash University Malaysia, Jalan Lagoon Selatan, 47500 Bandar Sunway, Selangor Malaysia; 4 Anqing Normal University, Anqing, China; 5 Mood Disorders Psychopharmacology Unit, Poul Hansen Family Centre for Depression, Toronto, Canada; 6 Department of Pharmacology and Toxicology, University of Toronto, Toronto, Canada; 7 Department of Psychiatry, University of Toronto, Toronto, Canada; 8 Brain and Cognition Discovery Foundation, Toronto, Canada; 9 Institute for Health Innovation and Technology (iHealthtech), National University of Singapore, Singapore, 117599 Singapore; 10 Department of Psychological Medicine, Yong Loo Lin School of Medicine, National University of Singapore, Singapore, 119228 Singapore; 11 Centre for Intelligent Signal and Imaging Research (CISIR), Universiti Teknologi PETRONAS, 32610 Seri Iskandar, Perak Malaysia

**Keywords:** Language, Prefrontal cortex

## Abstract

Living in high expressed emotion (EE) environments tends to increase the relapse rate in schizophrenia (SZ). At present, the neural substrates responsible for high EE in SZ remain poorly understood. Functional near-infrared spectroscopy (fNIRS) may be of great use to quantitatively assess cortical hemodynamics and elucidate the pathophysiology of psychiatric disorders. In this study, we designed novel low- (positivity and warmth) and high-EE (criticism, negative emotion, and hostility) stimulations, in the form of audio, to investigate cortical hemodynamics. We used fNIRS to measure hemodynamic signals while participants listened to the recorded audio. Healthy controls (HCs, $n=42$) showed increased hemodynamic activation in the major language centers across EE stimulations, with stronger activation in Wernicke’s area during the processing of negative emotional language. Compared to HCs, people with SZ ($n=41$) exhibited smaller hemodynamic activation in the major language centers across EE stimulations. In addition, people with SZ showed weaker or insignificant hemodynamic deactivation in the medial prefrontal cortex. Notably, hemodynamic activation in SZ was found to be negatively correlated with the negative syndrome scale score at high EE. Our findings suggest that the neural mechanisms in SZ are altered and disrupted, especially during negative emotional language processing. This supports the feasibility of using the designed EE stimulations to assess people who are vulnerable to high-EE environments, such as SZ. Furthermore, our findings provide preliminary evidence for future research on functional neuroimaging biomarkers for people with psychiatric disorders.

## Introduction

Schizophrenia (SZ) is a chronic and severe mental illness characterized by distortions in thinking, perception, emotions, and language, which has been affecting 20 million people worldwide^1^. People with SZ interpret reality abnormally and commonly experience hallucinations and delusions. They are associated with considerable disabilities to the extent that affect life, work, and social activities. The severity of SZ symptoms is typically assessed using Positive and Negative Syndrome Scale (PANSS)^2^. As SZ is a longstanding condition with the majority of people experiencing multiple relapses^3,4^, lifelong treatment is thus required, causing substantial personal, family, and healthcare burdens.

People with SZ living in the high expressed emotion (EE) environment have been found to be significantly associated with the recurrence of the illness^5–7^, posing a threat to their mental well-being and family harmony^8^. EE, in general, implies the attitudes shown by the family caregivers towards individuals with mental disorder. Family caregivers with high EE typically display hostility, criticism, and emotional over-involvement, whereas those with low EE portray warmth, positivity, and empathy^6^. A standard assessment, i.e., Camberwell Family Interview, is commonly used to assess EE level of the family caregivers in the absence of the patient^9^. In light of the high EE factor, a number of psychosocial intervention strategies, e.g., crisis management, emotional support, and education, have been provided to the family caregivers to reduce their distress and improve family-patient communication styles^6^.

SZ is currently treated with an individually tailored combination of psychotherapy and medicine (antipsychotic drugs)^10^. Along the pathway of discovering potential diagnosis and treatment in SZ, neuroimaging studies have been extensively conducted to investigate neural substrates of SZ symptoms^11–13^. Recent advances in neuroscience have shed some light on the structural and functional brain abnormalities in SZ^14,15^. One of the emerging non-invasive neuroimaging modalities, i.e., functional near-infrared spectroscopy (fNIRS), shows great potential to be developed as a diagnostic and neurotherapeutic tool for psychiatric disorders^16^. The measured fNIRS signals, i.e., oxygenated (HbO) and deoxygenated (HbR) hemoglobin, reflect the neurovascular coupling mechanisms occurring in the cerebral cortex^17^. Previous studies reported that HbO changes could differentiate a variety of psychiatric disorders during the performance of cognitive tasks^18,19^.

However, to the best of our knowledge, fNIRS has yet to be used for EE evaluation. At present, the neural substrates responsible for high EE remain poorly understood. A functional magnetic resonance imaging (fMRI) study examined EE using recorded speech containing critical or neutral comments, but in a small sample size of people with SZ (*n* = 11)^20^. There were a few fMRI studies assessed EE, but not on the people with SZ^21–23^. On the other hand, there were quite a large number of neuroimaging studies investigating the emotion processing of positive, neutral, and negative stimuli in SZ using faces, images, and voices^24–27^. This emphasizes the need of further investigation to understand neural substrates underlying high EE in SZ, which can be an opportunity to develop interventions for relapse prevention.

In this study, we design novel low- and high-EE stimulations that are closely matched to real-life scenarios. Targeting the frontal and left temporal cortices responsible for emotional language processing^28–30^, we aim to investigate the hemodynamic response reacting to different EE environments in people with SZ. A multi-channel fNIRS system is used to measure the hemodynamic signals due to its relatively lower cost, higher portability, and better balance between spatial and temporal resolution. Based on the studies examining emotion processing of different stimuli in SZ^24–27^, we hypothesize that (i) healthy controls (HCs) would show increased hemodynamic activity during emotional language processing with a larger increase in responding to high EE, (ii) people with SZ would exhibit reduced hemodynamic activity as compared to HCs during emotional language processing, and (iii) distinct differences in hemodynamic activity associated with the processing of high EE should be observed between HCs and people with SZ.

## Results

### Participant characteristics

Two HCs were removed due to technical errors during data collection. Another four HCs and six people with SZ were removed as they did not meet the requirements for channel confirmation in one of the region of interests (ROIs). A total of 96 corrupted trials (11.6%) from the remaining participants were discarded from the analysis. Table 1 summarizes the characteristics of the HCs (*n* = 42) and people with SZ (*n* = 41). People with SZ were older than HCs ($p<0.001$) and had fewer years of education than HCs ($p<0.001$). There was a significant difference in the sex between people with SZ and HCs ($p<0.05$).

**Table 1:** Descriptive demographic, clinical, and experimental characteristics of the groups.

	HC (${\varvec{n}}=42$)	SZ (${\varvec{n}}=41$)	Group differences
Age (years)	$19.4\pm 1.0$	$30.6\pm 5.3$	$t\left(81\right)=13.4, p<0.001$
Sex			
Male	$11 \left(26.2\%\right)$	$22 (53.7\%)$	${\chi }^{2}\left(2, n=83\right)=6.5, p<0.05$
Female	$31 \left(73.8\%\right)$	$19 (46.3\%)$	
Education (years)	$14.4\pm 1.0$	$10.8\pm 2.8$	$t\left(81\right)=7.9, p<0.001$
Age at onset (years)	–	$20.8\pm 4.3$	–
Duration of illness (years)	–	$9.7\pm 5.4$	–
PANSS score			
Positive scale	–	$15.3\pm 6.8$	–
Negative scale	–	$17.6\pm 5.8$	–
General psychopathology scale	–	$34.7\pm 10.9$	–
Number of noise-free channels			
mPFC	$8.7\pm 0.6$	$7.0\pm 2.0$	$t\left(81\right)=5.4, p<0.001$
Left IFG	$2.9\pm 0.$3	$2.8\pm 0.$4	$t\left(81\right)=0.9, p=0.35$
Left STG	$3.5\pm 0.6$	$3.6\pm 0.5$	$t\left(81\right)=0.5, p=0.62$
Number of noise-free EE trials			
Low EE	$4.3\pm 0.5$	$4.4\pm 0.5$	$t\left(81\right)=1.2, p=0.25$
High EE	$4.4\pm 0.7$	$4.5\pm 0.6$	$t\left(81\right)=1.0, p=0.33$

Data are expressed as mean $\pm$ SD.

Statistically significant at $p<0.05$ are shown in bold. Bonferroni correction was applied whenever applicable.

The mean and standard deviation (SD) of positive, negative, and general psychopathology scale scores calculated from PANSS scores were $15.3\pm 6.8$, $17.6\pm 5.8$, and $34.7\pm 10.9$, respectively. The number of noise-free channels between groups was significantly different in the medial prefrontal cortex (mPFC) ($p<0.001$), but not in the left inferior frontal gyrus (IFG) and left superior temporal gyrus (STG) ($p>0.05$). In addition, there were no significant differences in the number of noise-free low- and high-EE trials in within- and between-group analysis ($p>0.05$).

### Subjective rating of feeling

**Figure 1:**
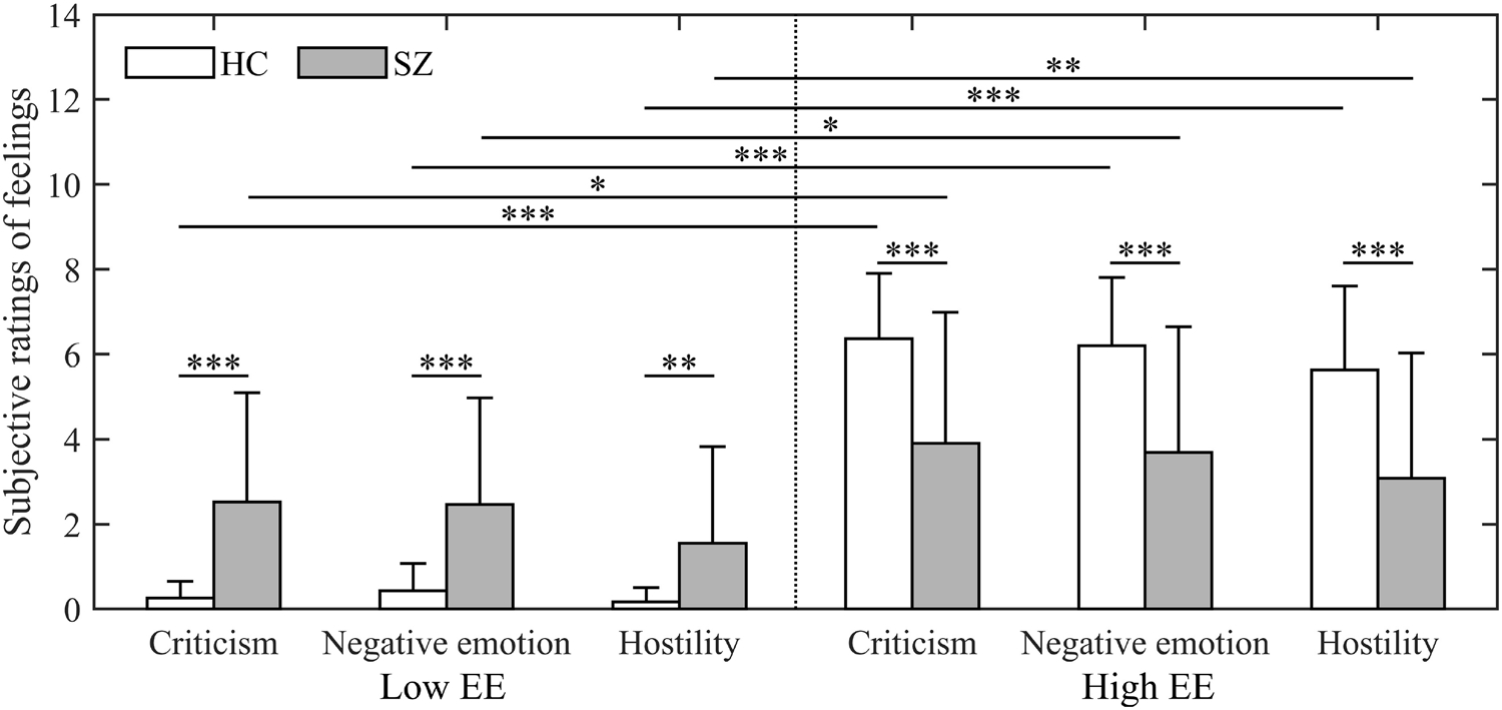
Subjective rating of feeling in HCs and people with SZ. Error bars indicate SD. Statistical significance with Bonferroni correction is shown: *$p<0.05$, **$p<0.01$, and ***$p<0.001$.

Figure 1 shows the subjective rating of feeling in HCs and people with SZ. Both groups had stronger feelings of criticism, negative emotion, and hostility during high-EE stimulations as compared to low-EE stimulations ($p<0.05$). Compared to HCs, people with SZ rated stronger feelings of criticism, negative emotion, and hostility for low EE ($p<0.01$) and weaker feelings for high EE ($p<0.001$).

### fNIRS signal change

**Figure 2:**
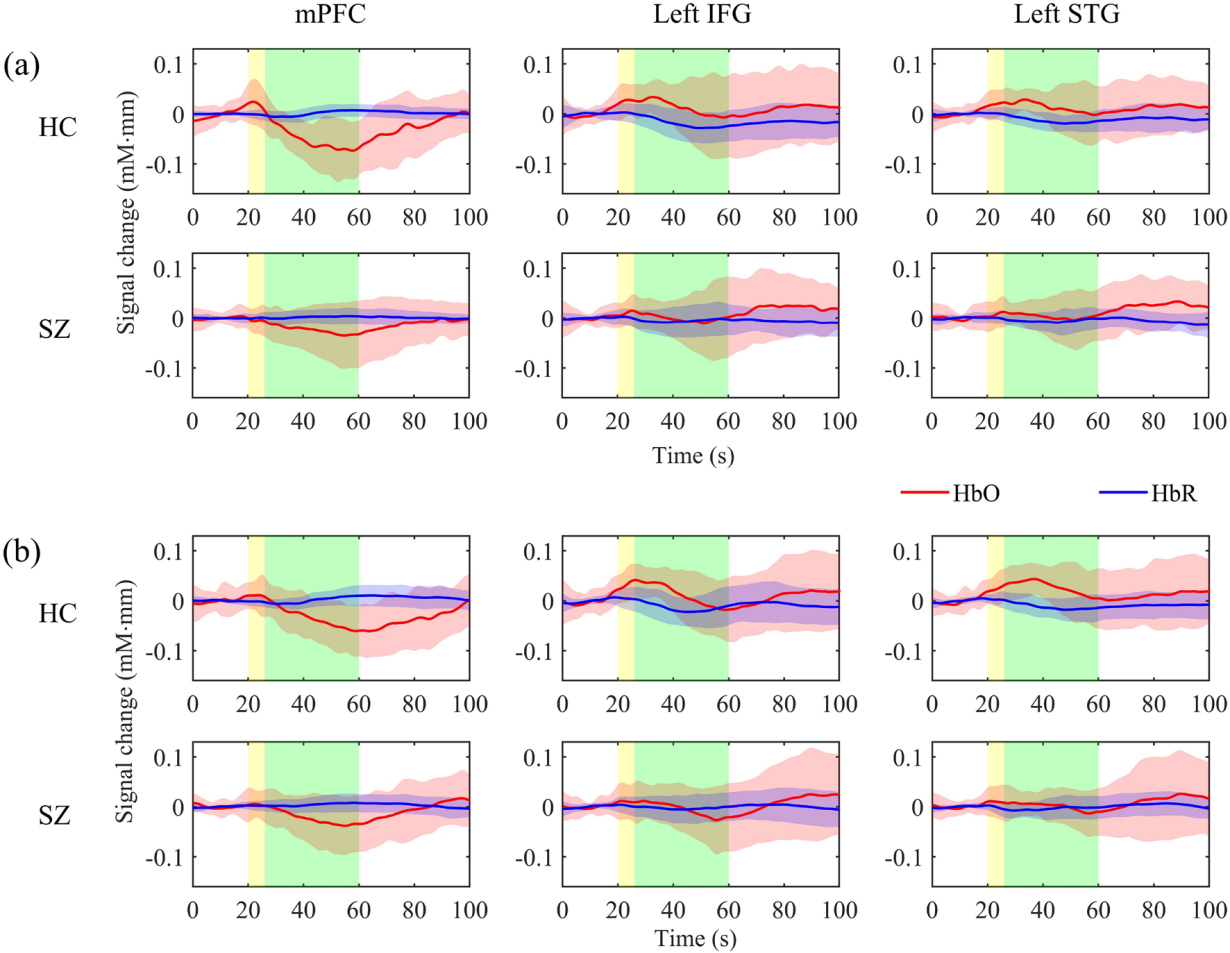
Grand average of time-series fNIRS signals for each ROI in HCs and people with SZ during (**a**) low- and (**b**) high-EE stimulations. The transparent shaded areas (red and blue colors) indicate SD at each time point. The transparent yellow and green shaded areas show the duration of listening to EE scenarios and dialogues, respectively.

**Figure 3:**
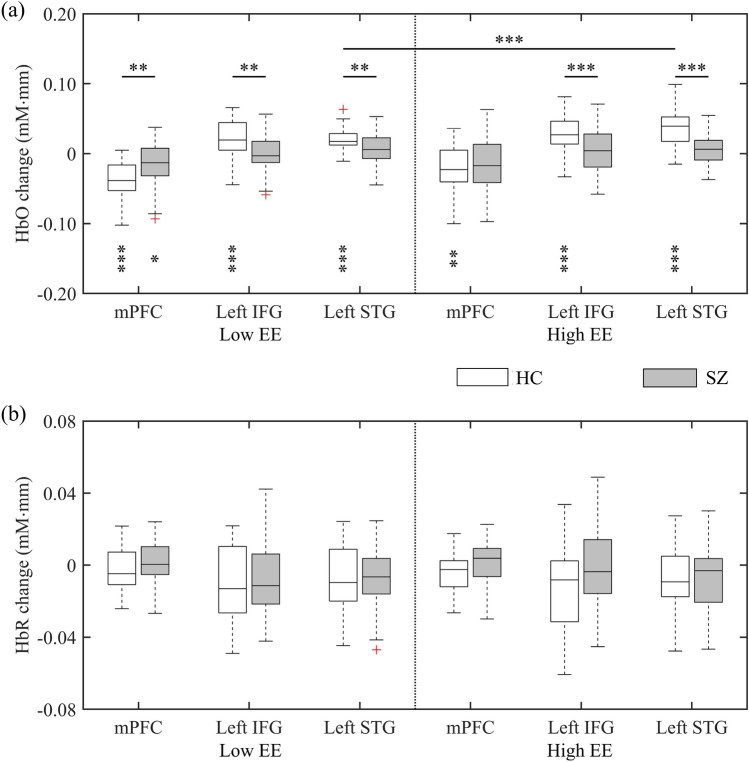
Visual representation of the hemodynamic response metric: (**a**) HbO and (**b**) HbR activation. Bonferroni-corrected one-sample *t*-test against zero was applied to each activation, where the significance levels (*) are placed at the bottom of the boxplots. In addition, significant pairwise comparisons obtained from the follow-up analysis of three-way mixed ANOVA are shown. *$p<0.05$, **$p<0.01$, and ***$p<0.001$ indicate statistical significance.

Figure 2 shows the grand block-average time-series fNIRS signals of mPFC, left IFG, and left STG for each group and EE stimulation. Figure 3 displays the visual representation of the hemodynamic response metric, i.e., the brain activation. During low-EE stimulations, HCs showed a significant HbO increase from the baseline in the left IFG ($p<0.001$) and left STG ($p<0.001$), but not in people with SZ ($p>0.05$). Both HCs ($p<0.001$) and people with SZ ($p<0.05$) showed a significant HbO decrease from the baseline in the mPFC. Similarly, during high-EE simulations, HCs showed a significant HbO increase from the baseline in the left IFG ($p<0.001$) and left STG ($p<0.001$), but not in people with SZ ($p>0.05$). In addition, HCs showed a significant HbO decrease from the baseline in the mPFC ($p<0.01$), but not in people with SZ ($p>0.05$). None of the HbR changes were significant from the baseline ($p>0.05$).

A three-way mixed analysis of variance (ANOVA) was applied to understand the effects of group (HCs and people with SZ), ROI (mPFC, left IFG, and left STG), and EE (low and high) on HbO activation. There was no significant three-way interaction effect between group, ROI, and EE $\left[F\left(1.7, 138.7\right)=1.2, p=0.30, {\eta }_{p}^{2}=0.014\right]$. The two-way interaction effect was significant (i) between group and ROI $\left[F\left(1.7, 138.9\right)=24.8, p<0.001, {\eta }_{p}^{2}=0.234\right]$ and (ii) between group and EE $\left[F\left(1, 81\right)=4.4, p<0.05, {\eta }_{p}^{2}=0.052\right]$, but was not significant between ROI and EE $\left[F\left(1.7, 138.7\right)=0.9, p=0.39, {\eta }_{p}^{2}=0.011\right]$.

Based on the follow-up evaluation, HCs had a significant simple main effect of EE in the left STG $\left[F\left(1, 41\right)=21.3, p<0.001, {\eta }_{p}^{2}=0.342\right].$ A simple main effect of group was significant in all three ROIs: mPFC $\left[F\left(1, 81\right)=9.4, p<0.01, {\eta }_{p}^{2}=0.103\right]$, left IFG $\left[F\left(1, 81\right)=10.6, p<0.01, {\eta }_{p}^{2}=0.115\right]$, and left STG $\left[F\left(1, 81\right)=10.6, p<0.01, {\eta }_{p}^{2}=0.115\right]$ at low EE. Meanwhile, at high EE, a simple main effect of group was significant in the left IFG $\left[F\left(1, 81\right)=12.1, p<0.001, {\eta }_{p}^{2}=0.130\right]$ and left STG $\left[F\left(1, 81\right)=37.4, p<0.001, {\eta }_{p}^{2}=0.316\right]$, but not in the mPFC $\left[F\left(1, 81\right)=0.5, p=0.47, {\eta }_{p}^{2}=0.006\right]$. On the other hand, two- and three-way interactions between group, ROI, and EE on HbR activation were not significant ($p>0.05$).

### Correlation

Table 2 displays the correlations between the subjective rating of feeling and fNIRS activation in HCs and people with SZ. None of them were significantly correlated ($p>0.05$). Table 3 presents the correlations between the PANSS score and fNIRS activation in people with SZ. A significant moderate negative correlation of negative syndrome scale score with HbO activation was observed in the left STG ($r=-0.482, p<0.05$) at high EE. None of the PANSS scores significantly correlated with HbR activation ($p>0.05$).

**Table 2:** Correlation coefficient $r$ between the subjective rating of feeling and fNIRS activation.

EE	Group	Subjective rating of feeling	HbO activation			HbR activation		
			mPFC	Left IFG	Left STG	mPFC	Left IFG	Left STG
Low	HC	Criticism	− 0.183^●^	− 0.001^●^	− 0.204^●^	0.221^●^	− 0.293^●^	− 0.049^●^
		Negative emotion	0.121^●^	0.155^●^	− 0.002^●^	0.099^●^	− 0.285^●^	− 0.079^●^
		Hostility	0.035^●^	0.075^●^	− 0.125^●^	0.042^●^	− 0.334^●^	− 0.151^●^
	SZ	Criticism	0.106^●^	0.031^●^	− 0.146^●^	0.148	− 0.179^●^	− 0.103
		Negative emotion	0.078	0.225	0.081	0.185	− 0.166^●^	− 0.127
		Hostility	0.060^●^	0.050^●^	0.012^●^	− 0.050^●^	− 0.131^●^	− 0.133^●^
High	HC	Criticism	− 0.152	− 0.062	− 0.118	− 0.075^●^	− 0.054	− 0.037^●^
		Negative emotion	− 0.122^●^	− 0.209^●^	− 0.125^●^	− 0.007^●^	− 0.152^●^	− 0.194^●^
		Hostility	− 0.160	− 0.045	− 0.220	− 0.054	− 0.022	− 0.020
	SZ	Criticism	0.123^●^	0.028	0.026	0.066	0.070	0.137
		Negative emotion	0.184	0.037^●^	0.062	0.181	0.111	0.240
		Hostility	0.070	0.054	0.090	0.223	0.098	0.280

^●^Spearman’s rank-order correlation was applied due to violation of bivariate normality.

**Table 3:** Correlation coefficient $r$ between the PANSS score and fNIRS activation in people with SZ.

EE	PANSS score	HbO activation			HbR activation		
		mPFC	Left IFG	Left STG	mPFC	Left IFG	Left STG
Low	Positive scale	− 0.094	0.133	− 0.038	− 0.125	0.128	0.086
	Negative scale	0.027	− 0.016	− 0.221	− 0.013	0.249	0.171
	General psychopathology scale	− 0.125	− 0.064	− 0.054	− 0.043	0.128	− 0.058
High	Positive scale	− 0.096	− 0.212	− 0.013	−0.187	0.076	− 0.160
	Negative scale	− 0.053	− 0.394	− 0.482*	0.239	0.052	0.121
	General psychopathology scale	0.295	− 0.431	− 0.174	− 0.216	0.023	− 0.046

*Statistical significance with Bonferroni correction, $p<0.05$.

## Discussion

In this study, we designed novel low- and high-EE stimulations to investigate the cortical hemodynamics in SZ. Judging from the self-rating scores, HCs were able to clearly distinguish the presence of criticism, negative emotion, and hostility between low- and high-EE stimulations as compared to people with SZs. This could imply that our designed tasks meet the requirements of low- and high-EE environments^6^. In general, increased brain activity during language processing is commonly observed in the major language centers, i.e., Broca’s and Wernicke’s areas^31–33^. These two areas are typically located in the left IFG and left STG of the right-handed people, respectively^28,34^. Likewise, in HCs, we found that the HbO signal increased in these two areas when responding to low- and high-EE dialogues, indicating the occurrence of neural activity responsible for speech comprehension.

Notably, HCs showed greater HbO activation in the left STG when responding to high-EE dialogues, reflecting increased attention and demand to process negative emotional contents. A mixture of findings was reported in the previous fMRI studies, where the brain activity in Broca’s and Wernicke’s areas was found to increase^31,35–37^ or have no significant difference^38–40^, when processing or expressing negative emotional prosody as compared to the positive ones. Our findings suggest that Wernicke's area is sensitive to the emotional valence of language and its activation may be modulated by emotional contents.

People with SZ did not exhibit increased HbO signal in the major language centers when processing low- and high-EE dialogues. However, it is worth noting that HbO activation was higher in HCs as compared to people with SZ, regardless of emotional content. Similar findings were reported in fMRI^12,41,42^ and fNIRS^18,19,43^ studies comparing the brain activity in HCs and people with psychiatric disorders across a wide range of cognitive tasks, suggesting that the regional brain volume loss in SZ may play a part^44,45^. These findings also indicate that people with SZ may have alteration and disruption in the neural processing of emotional language.

The mPFC is known to play a crucial role in emotion regulation by exerting inhibitory control over the amygdala (not measurable by fNIRS), i.e., a key region responsible for emotion processing^46–48^. Interestingly, HCs showed decreased HbO signal in the mPFC regardless of emotional contents. Similar findings were reported in an fNIRS study utilizing comedy, horror, and landscape movies as emotion stimulations^49^. Such a decrease from the baseline is known as brain deactivation, which corresponds to part of the so-called default mode network having high baseline activity at rest but deactivating across a wide range of cognitive tasks^50^.

People with SZ, however, did not exhibit decreased HbO signal in the mPFC during high-EE stimulations. Compared to HCs, a weaker mPFC deactivation was observed in people with SZ during low-EE stimulations. In an fMRI study, people with SZ showed weaker mPFC deactivation while passively viewing faces, regardless of the emotional contents^51^. In addition, a number of fMRI studies found that people with SZ failed to deactivate mPFC during the performance of working memory tasks^52–54^. Taking all these into interpretations, our results suggest that the default mode network in SZ is likely to be dysfunctional. On the other hand, HbR activation was not differ across groups and EE stimulation, probably due to its lower sensitivity in fNIRS recordings^55^.

Notably, the negative syndrome scale score was negatively associated with HbO activation in Wernicke’s area during high-EE stimulations. This indicates that people with SZ, in particular, those with higher level of severity in negative symptoms, i.e., a loss of normal function related to motivation and interest, tend to have much weaker HbO activation when processing negative emotional language. This could potentially link to the higher relapse rate in people with SZ exposed to high-EE environments^5–7^. Previous studies also reported significant correlations between PANSS score and brain activation in SZ over a number of cognitive tasks^56–58^. Moreover, cognitive impairment in SZ is typically associated with the severity in negative symptoms^59,60^.

This study has a number of limitations. Due to the COVID-19 pandemic and restriction of human movements, we were not able to recruit age-, gender-, and education-matched HCs. We understand that the language and emotion processing abilities in humans change across the lifespan, where children undergo significant development during early childhood and continue to mature into adolescence and adulthood^61,62^. This brings complexity to the analysis of emotional language processing associated with age, gender, and education. Taking into account that all participants had a minimum of nine compulsory education years, we used simple vocabulary in EE stimulation designs to ease language comprehension and minimize educational influence.

In addition, we understand that the experimental design can result in order effects as the EE stimulations were not counterbalanced. However, judging from the HCs findings in the aspects of subjective rating and HbO activation, the order effects should be at a minimal level. Furthermore, due to the limitation of our fNIRS hardware, we were not able to assess the right temporal and lateral frontal cortices. There were studies reporting the involvement of these regions during emotional language processing^63–66^. Future works should consider (i) recruiting demographic-matched HCs, (ii) counterbalancing the experimental design, (iii) expanding fNIRS recordings to other relevant brain regions, and (iv) translating the protocol to other languages to examine its repeatability and reliability.

In conclusion, the proposed EE stimulations could be used to assess people who are vulnerable to high EE environments, such as SZ and other psychiatric disorders. This is the first time an fNIRS protocol, incorporated with well-designed EE stimulations, was applied to HCs and people with SZ. The findings from this study provide preliminary evidence for future research on functional neuroimaging biomarkers for people with psychiatric disorders. It could potentially establish the groundwork for understanding the psychopathology in SZ using the combination of EE stimulations with neuroimaging tools like fNIRS. Taking into account the distinct patterns of HbO activation, our findings can be adopted as part of the clinical profiles in personalized medicine and computational algorithms to predict prognosis in people suffering from SZ.

## Methods

### Participants

This study involved a total of 48 HCs (*n* = 35 females) and 47 people with SZ (*n* = 20 females), all of whom were Mandarin-speaking, right-handed, aged between 18 and 42 years old. HCs were recruited from the community in Huaibei, China. None of the HCs had a history of serious head injury, neurological, or psychiatric illness. People with SZ were recruited from the Huaibei Mental Health Center, Huaibei, Anhui Province, China, and they were hospitalized at the time of the recruitment. The recruitment and data collection periods were between November 1 and December 15, 2021. In order to detect an effect of partial eta squared ${n}_{p}^{2}=0.06$ (equivalent to Cohen’s *d* $=0.5$, medium effect) with 80% power in repeated measure ANOVA (within-between interaction: two groups, two measurements, significant level $=0.05$), G*Power suggested a total sample size of 34 participants, corresponding to 17 participants per group^67^.

Prior to the study, the diagnosis of SZ was established by psychiatrists based on the Diagnostic and Statistical Manual of Mental Disorders, Fifth Edition (DSM-5)^68^. In this study, SZ was further assessed based on a structured clinical interview using PANSS^2^. All of them had paranoid SZ and were prescribed a variety of antipsychotic medications, such as risperidone, sodium valproate, clozapine, sulpiride, aripiprazole, quetiapine, perphenazine, and olanzapine. Study details were fully explained to potential participants and their written informed consent was obtained. People with SZ were informed that participation in this study was voluntary. Agreement or refusal to participate in this study would not have any effect on their clinical care and management. The authors assert that all procedures contributing to this work comply with the ethical standards of the relevant national and institutional committees on human experimentation and with the Helsinki Declaration of 1975, as revised in 2008. All procedures involving human subjects/patients were approved by Huaibei Normal University Institutional Review Board (Protocol number HBU-IRB-2021-001).

### Expressed emotion stimulation

The native language, i.e., Mandarin, was used in the experimental design to facilitate understanding and comprehension. Considering the difference in living environments of HCs and people with SZ, we systematically prepared a total of five scenarios for each HC and people with SZ. Each scenario was equipped with two designed dialogues to represent low- and high-EE environments, respectively. Low-EE dialogues contain positivity and warmth, whereas high-EE dialogues encompass criticism and hostility. An expert was assigned to express the low- and high-EE dialogues in positive and aggressive tones, respectively. All scenarios and dialogues were recorded in high quality.

The participants sat comfortably during the experiment and wore earphones. A typical block design was used in this study, where a trial includes pre-stimulation, stimulation, and post-stimulation periods^69^. During the stimulation period, participants listened to audio recordings of the scenario (~ 6 s) and followed by EE dialogues (~ 34 s). During 20-s pre-stimulation and 40-s post-stimulation periods, participants were asked to remain seated and stay relaxed. The duration of pre- and post-stimulation periods was assessed with the baseline state detection approach in a pilot study, where a total of 60-s duration was found to be sufficiently long for the evoked hemodynamic response to return to baseline^70^.

**Figure 4:**
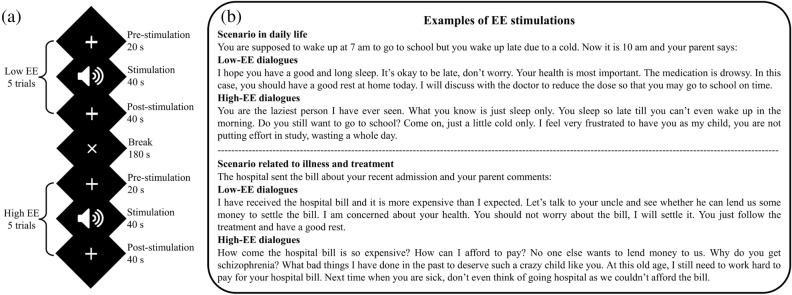
EE stimulation: (**a**) the experimental flow and (**b**) examples of EE scenarios and dialogues (translated to English).

Figure 4 shows the experimental flow and examples of EE scenarios and dialogues. Considering the intense emotional disturbances that may trigger people with SZ in high-EE environments, we chose not to begin the experiment with high-EE stimulations. Thus, participants completed the first session consists of five low-EE stimulations and followed by a short 3-min break before continuing the second session consists of five high-EE stimulations. The participants were briefed on the experimental flow, but they were blindfolded on the task nature to avoid bias and expectation. The participants were instructed to position themselves in each dialogue to stimulate EE environments.

### Subjective rating of feeling

The subjective rating of feeling is typically collected in emotion-based neuroimaging studies^49,71^. At the end of the experiment, a subjective questionnaire form containing the texts of the scenarios and EE dialogues was provided to each participant to rate his/her feeling in the aspect of criticism, negative emotion, and hostility on a 9-point scale (0 = no feeling, 8 = strongest feeling). The terms criticism, negative emotion, and hostility were explained with examples to avoid misunderstanding. The subjective rating of feeling was not conducted during fNIRS recordings to prevent unwanted motions and unwanted brain activities from affecting the signal quality and interpretation.

### fNIRS data acquisition

A 10-Hz sampling-rate wearable fNIRS device (Brite 24; Artinis Medical Systems, Netherlands) consisting of 10 light sources and 8 detectors was used to record hemodynamic signals at frontal and left temporal cortices. A total of 22 measurement channels were available with the 30-mm source-detector distance configurations. The light sources emitted near-infrared light at wavelengths of 762.5 and 841 nm.

**Figure 5:**
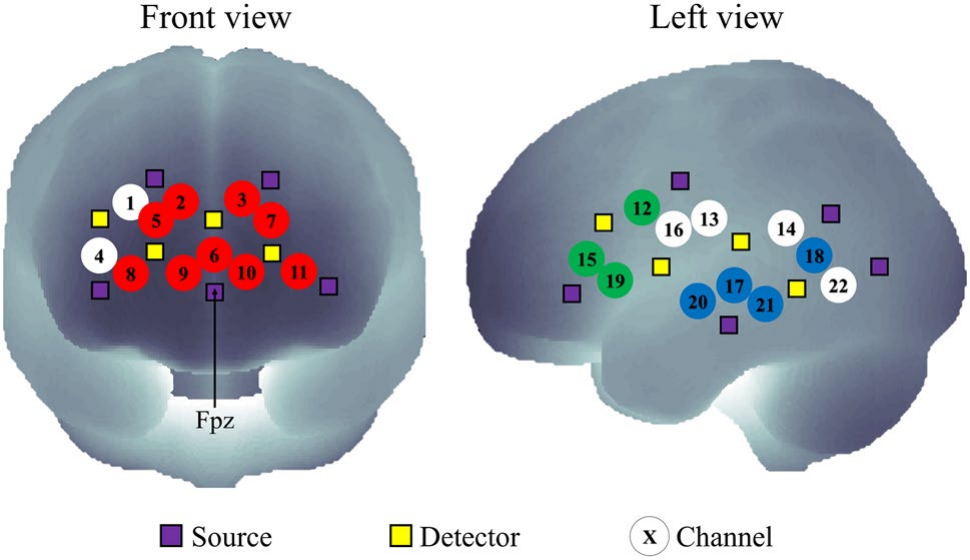
Spatial registration of fNIRS channels onto a standard brain template in MNI space. Channels were classified into three ROIs: mPFC (red-colored channels), left IFG (green-colored channels), and left STG (blue-colored channels). White-colored channels were not located in ROIs and hence they were excluded from the analysis.

Based on the 10–20 international system^72^, the light sources and detectors of our fNIRS system are provided with Montreal Neurological Institute (MNI) coordinates for spatial registration of channel locations with NIRS-SPM toolbox^73^. Figure 5 shows the registered channels and the placements of light sources and detectors using Fpz as the reference. The Brodmann area (BA) number of each channel was determined according to Rorden’s brain atlas from the MNI coordinates (Supplementary Table S1)^74^.

Previous studies reported the involvement of mPFC in processing and regulating emotion^29,30^. The major language centers, i.e. Broca’s and Wernicke’s areas, responsible for speech production and comprehension, are mainly located in the left IFG and left STG of the right-handed people^28,34^, respectively. Hence, channels were categorized into three ROIs: mPFC (BAs 9–11), left IFG (BAs 44, 45), and left STG (BA22). Channels 1 and 4 were excluded to ensure a symmetrical number of channels between left and right mPFC. As there were a limited number of channels registered in the left IFG and left STG, we considered them as part of the ROIs when their respective BA numbers overlapped at least 20%.

### fNIRS signal analysis

MATLAB (MathWorks Inc., United States) was primarily used for data analysis. Prior to analysis, about 5% of the total number of channels was eliminated due to the poor signal-to-noise ratio (SNR; power ratio of 0.005–0.2 Hz to 4–5 Hz less than 20 dB). The light intensity data were transformed into the optical density data and were followed by motion artifacts correction with a temporal derivative distribution repair algorithm on a channel-by-channel basis^75^. Modified Beer–Lambert law was applied to obtain time-series HbO and HbR signals in the unit of a millimolar millimeter (mM·mm)^76^. Third-order Butterworth bandpass filter with cutoff frequencies between 0.005 and 0.2 Hz was used to remove baseline drift and high-frequency noise.

The detailed analysis was focused on the ROIs as our interest was to investigate task-related fNIRS signals. A comprehensive description of removing non-active channels and corrupted EE trials in each ROI is provided in Supplementary Method and Supplementary Fig. S1. Channel-wise signals were cut from the beginning of pre-stimulation period to the end of post-stimulation period. The noise-free trial-wise signals were obtained from each channel, signal type, and EE type, which was then baseline fitted by the 20-s average amplitude of pre-stimulation.

Grand task-related fNIRS signals in each ROI were obtained by averaging the respective trial-wise signals. Considering the peak activation factor, the average amplitude of grand HbO/HbR signals from 6 to 26 s after task onset was used to derive the brain activation, which is said to be indicative of both the magnitude and direction of task-related hemodynamic response^55^.

### Statistical analysis

SPSS Statistics (IBM, Armonk, NY) was used to perform statistical analysis. The differences between groups on categorical variables were determined using the chi-square test, whereas the *t*-test was applied to continuous variables. The categorical variable is gender, whereas continuous variables are age, education, subjective rating, and brain activation. All tests were two-tailed with a significance level of *p* < 0.05. Data are expressed as mean and SD. Bonferroni correction was applied whenever necessary for multiple comparisons.

A three-way mixed ANOVA followed by simple effects analysis and Bonferroni post hoc test was carried out to study the interaction between the group, ROI, and EE on brain activation. Greenhouse–Geisser correction was applied if Mauchly’s test of sphericity indicates any violation of sphericity. The effect size was determined using the partial eta squared $({n}_{p}^{2})$.

In addition, Pearson’s correlation analysis was performed to examine the associations between (i) the subjective rating of feeling and brain activation in HCs and people with SZ, and (ii) the PANSS score (positive, negative, and general psychopathology scales) and brain activation in people with SZ. Spearman’s rank-order correlation analysis was applied if Mardia's multivariate skewness and kurtosis test indicates any violation of bivariate normality.

## Supplementary Information

Supplementary Information.

## Data Availability

The datasets generated and analyzed during the current study are not publicly available due to privacy and ethical restrictions but are available from the corresponding author on reasonable request.
