# Supplementary material for: An fNIRS investigation of novel expressed emotion stimulations in schizophrenia
Source: Sci Rep. 2023 Jul 10;13:11141. doi: 10.1038/s41598-023-38057-1 (PMC10333297; doi:10.1038/s41598-023-38057-1)
Supplement: Supplementary file 1 — Supplementary Information. [file 41598_2023_38057_MOESM1_ESM.docx]

**Supplementary Material**

**Table S1:** The MNI coordinates with BA numbers and overlapping percentages for each fNIRS channel.

| **Channel** | **MNI coordinates** | | | **BA number** | **Percentage (%)** |
| --- | --- | --- | --- | --- | --- |
|  | $\boldsymbol{x}$ **(mm)** | $\boldsymbol{y}$ **(mm)** | $\boldsymbol{z}$ **(mm)** |  |  |
| 1 | 28.7 | 60.7 | 27.7 | 46  10  9 | 55  38  7 |
| 2 | 10.7 | 67 | 29.3 | 10  9 | 91  9 |
| 3 | -9.3 | 65.3 | 30.3 | 10  9 | 81  19 |
| 4 | 42.7 | 60.7 | 7.7 | 10  46 | 59  41 |
| 5 | 20.7 | 68 | 23.7 | 10  46 | 97  3 |
| 6 | –0.3 | 68 | 10 | 10 | 100 |
| 7 | –18.7 | 66.7 | 24.3 | 10  46  9 | 92  6  2 |
| 8 | 34.3 | 67 | 3.3 | 10  11 | 69  31 |
| 9 | 12.3 | 74 | 4.7 | 10  11 | 94  6 |
| 10 | –12.7 | 74 | 4.7 | 10  11 | 88  12 |
| 11 | –33.7 | 65 | 4 | 10  11 | 80  20 |
| 12 | –62.3 | 7.7 | 28.3 | 6  44  43  4 | 60  20  17  3 |
| 13 | –68 | –15.7 | 25.7 | 43  2  22  48  1 | 43  32  12  11  2 |
| 14 | –68 | ­–41.7 | 21.7 | 22  48  42  40 | 68  26  3  3 |
| 15 | –57 | 29.3 | 10.3 | 45  44  48 | 91  5  4 |
| 16 | –65 | 0 | 21 | 43  6  48  22 | 63  31  4  2 |
| 17 | –71 | –25.3 | 2.7 | 21  22 | 60  40 |
| 18 | –67 | –52.3 | 14.3 | 22  21  37 | 49  32  19 |
| 19 | –58 | 21.3 | 5.3 | 45  48  38  44  6 | 35  31  16  14  4 |
| 20 | –68 | –11.3 | –2.7 | 21  22  48 | 60  35  5 |
| 21 | –71 | –35.3 | –3.7 | 21  22  20 | 62  20  18 |
| 22 | –65 | –59.3 | 2.3 | 37  21 | 86  14 |

**Supplementary Method**

It was critical to ensure that the channels in ROIs were not contaminated by noisy or non-activated signals. Figure S1 shows the flowchart of fNIRS signal preprocessing to channel confirmation for each ROI. We applied a simple theory to confirm the quality of the signals in each ROI, i.e., the time-series HbO signal of the channels should be positively correlated with each other^1^. Pearson’s correlation was conducted between each channel with all other respective channels in each ROI. The average correlation was computed for each channel. A channel was removed from the ROI if its average correlation was less than 0.5. The 0.5 threshold corresponds to the “moderate” correlation strength^2^. The steps were repeated till every channel in the ROI satisfied the condition. If only a single channel remained in the ROI, the participant would be removed from further analysis. This also implies that each ROI needs to have at least two channels for confirmation.

In addition, we identified those HbO deviations (without temporal derivative distribution repair algorithm) that exceed the 0.05-mM·mm threshold within two consecutive sampling points as severe motion artifacts^3^. The trials were considered corrupted when more than 10% of the sampling points over the task duration were identified as severe motion artifacts.


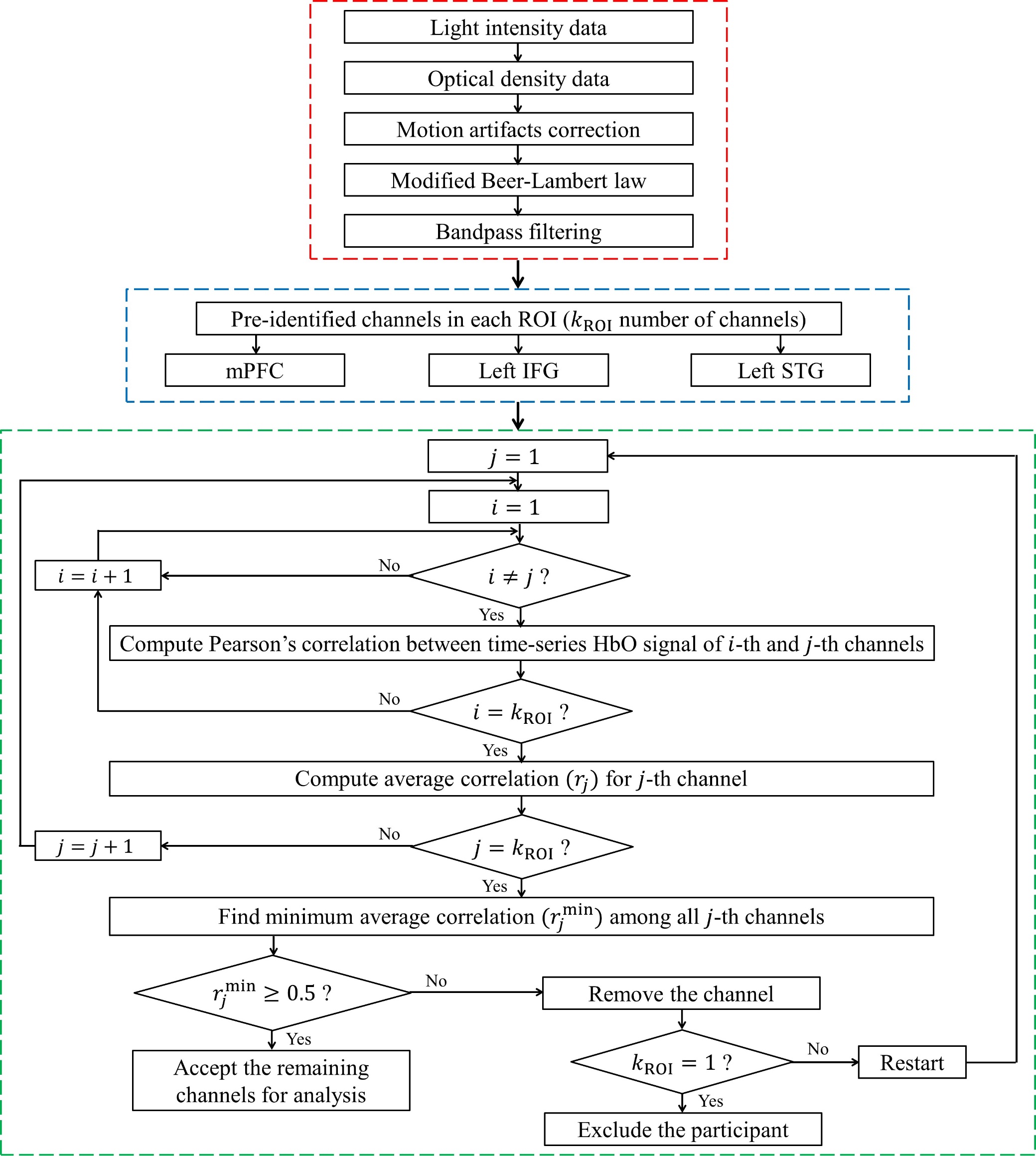


**Figure S1:** The flowchart of fNIRS signal preprocessing to channel confirmation for each ROI.

**References**

1 Sato, H. *et al.* A NIRS–fMRI investigation of prefrontal cortex activity during a working memory task. *Neuroimage* **83**, 158-173 (2013).

2 Akoglu, H. User's guide to correlation coefficients. *Turkish journal of emergency medicine* **18**, 91-93 (2018).

3 Sutoko, S. *et al.* Tutorial on platform for optical topography analysis tools. *Neurophotonics* **3**, 010801 (2016).
